# *Calotropis procera* (leaves) supplementation exerts curative effects on promoting functional recovery in a mouse model of peripheral nerve injury

**DOI:** 10.1002/fsn3.2455

**Published:** 2021-07-09

**Authors:** Shamaila Zafar, Azhar Rasul, Javed Iqbal, Haseeb Anwar, Ali Imran, Farhat Jabeen, Asghar Shabbir, Rabia Akram, Javeria Maqbool, Faiqa Sajid, Muhammad Umair Arshad, Ghulam Hussain, Saiful Islam

**Affiliations:** ^1^ Neurochemicalbiology and Genetics Laboratory (NGL) Department of Physiology Faculty of Life Sciences Government College University Faisalabad Pakistan; ^2^ Department of Zoology Faculty of Life Sciences Government College University Faisalabad Pakistan; ^3^ Department of Neurology Allied Hospital Faisalabad Medical University Faisalabad Pakistan; ^4^ Institute of Home and Food Sciences Government College University Faisalabad Pakistan; ^5^ Department of Biosciences COMSATS Institute of Information Technology Islamabad Pakistan; ^6^ Institute of Nutrition and Food Science University of Dhaka Dhaka Bangladesh

**Keywords:** arylesterase, paraoxonase‐1, ARE, peripheral nerve injury, TOS, PNI, sciatic functional index, PON‐1, total antioxidant capacity, SFI, total oxidant status, TAC

## Abstract

Peripheral nerve injuries are among those complicated medical conditions, which are still waiting for their highly effective first‐line therapies. In this study, the role of *Calotropis procera* crude leaves was evaluated at different doses for their effectiveness in improving functional recovery following sciatic nerve injury‐induced in the mouse model. Thirty‐two healthy albino mice were divided into four groups as Normal chow group (control, *n* = 8) and *C. procera* chow groups (50 mg/kg (*n* = 8), 100 mg/kg (*n* = 8) and 200 mg/kg (*n* = 8)). Behavioral analyses were performed to assess and compare improved functional recovery along with skeletal muscle mass measurement in all groups. Serum samples were analyzed for oxidative stress markers. Results showed that *C. procera* leaves at dose‐dependent manner showed statistically prominent (*p* < .05) increase in sensorimotor functions reclamation as confirmed by behavioral analyses along with muscle mass restoration and prominent decline in TOS and momentous increase in TAC along with the augmented activity of antioxidative enzymes.

## Graphical abstract

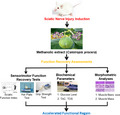

## 1 INTRODUCTION

Nerves, being the delicate long bundles of thousands of axons, are more prone to be injured following any type (either endogenous or exogenous) of the traumatic situation (Ring, 2013). Thus, peripheral nerve injury (PNI) is one of those commonly faced medical situations which are still completely incurable (Palispis & Gupta, 2017). Although such injuries are not life‐threatening (Warner et al., 2020) yet severe consequences in the form of life‐long disability and physical dependency always compel the researchers to seek such therapeutic means which could fully heal these injuries and save the patients from inevitable life‐long suffering of disability.

Upon the nerve being damaged, a cascade of pathological events including demyelination, inflammation, phagocytosis, and oxidative stress begins that abruptly stops the conduction of impulses to its respective organ/muscle and initiates nerve degeneration. It results in loss of sensory as well as motor functions (Weng et al., 2018). Mild to moderate type of nerve injuries have the capability to self‐heal and regenerate over time though their speed to regenerate is much slower that it takes months or even years to reinnervate the target tissue (Houschyar et al., 2016). Ultimately, denervated muscle starts shrinking over time. This muscular dystrophy leads to permanent sensorimotor loss (Imran et al., 2019; Rasul et al., 2019). Although a variety of therapeutic options have been adopted by physicians yet attaining fully functional recovery is still a challenge to be addressed (Hussain et al., 2020). In this regard, noticeable attention has been paying toward considering such therapeutically effective, easily accessible, and affordable agents capable enough to completely reclaim the sensorimotor functional loss by reducing the time acquired for axonal regeneration and preventing target tissue/muscle from being atrophied (Hussain, Rasul, et al., 2018; Hussain, Zhang, et al., 2018).

Local flora is highly enriched with naturally occurring therapeutically effective compounds that possess a long history of being used as therapeutic agents in various diseases (Hussain, Rasul, et al., 2018; Hussain, Zhang, et al., 2018). Exploration of such compounds would provide a promising alternative remedy for patients. *Calotropis procera* (*C. procera*) is a tropical plant growing widely across the world. It belongs to the family Apocynaceae and is commonly known as Mudar, Rubber Bush, or Apple of Sodom (Mali et al., 2019). It is well known as an Ayurvedic plant because of being used as a part of several traditional medicines to heal various diseases (Parihar & Balekar, 2016). All parts of this plant (such as roots, leaves, flowers, latex) are found to exhibit enormous health‐related properties such as anthelmintic, hepatoprotective, antitumor, antimicrobial, antioxidant, anti‐inflammatory, antipyretic, analgesic, anti‐angiogenic, antidiabetic, antifertility and, anticonvulsant activities (Mali et al., 2019; Mali et al., 2019; Parihar & Balekar, 2016). A recent study recommends the use of *C. procera* leaves extract as an alternative milk coagulant because of the presence of the calotropin enzyme in it and there is no toxicity found even at higher doses (Abebe & Emire, 2020). This plant has also been found to be associated with neuroprotection in neurodegenerative diseases, that is, Alzheimer's disease by attenuating the accumulation of amyloid‐beta proteins in neuronal tissues (Paul et al., 2018). Crude leaves powder has been documented to be used relieving joint pain and reducing inflammation (Meena et al., 2011). These pharmacological activities are considered to be due to the presence of highly effective phytoconstituents such as cardenolides, steroids, tannins, glycosides, phenols, terpenoids, sugars, flavonoids, alkaloids, and saponins in this plant (Al‐snafi, 2015). Thus, based upon the available data, *C. procera* can be ascribed as a good candidate for being a neuroprotective agent. But, up to now, the data regarding its nerve regenerative activity is quite limited. Therefore, we took advantage of our previously developed model of sciatic nerve injury to evaluate the role of *C. procera* leaves in escalating neuroregenerative processes.

## 2 MATERIALS & METHODS

### 2.1 Experimental animals

Healthy male albino mice (BALB/C) of an average age (8–10 weeks) and bodyweight 25–35 g were purchased from the animal housing facility of the Department of Physiology, Government College University Faisalabad. All animals were housed as 1 animal per cage (plastic rodent cage). Housing specification that is, room temperature of 23–27°C, provision of 12 hr light and 12 hr dark cycle, optimal humidity, and ad libitum supply of diet and drinking water were ensured during the period of acclimatization (1 week) and the whole experiment. All behavioral observations as well as other experiments were conducted during the light cycle.

### 2.2 Plant material gathering and processing

Fresh *C. procera* leaves were collected from the peripheral areas of Faisalabad in the spring season and were identified by the Department of Botany, Government College University, Faisalabad. These leaves were washed, shade dried, and then ground into fine powder by the mill. The powdered leaves were sieved via sieve no. 60 before mixing into the rodent diet. The leaves powder was mixed in a daily rodent diet at three different doses, that is, 50, 100, and 200 mg/kg body weight.

### 2.3 Surgical induction of Sciatic nerve lesion

The experimental mice, after been acclimatized for a week, were underwent+a surgical procedure to induce nerve lesion. They were anesthetized with deep anesthesia, that is, a mixture of Ketamine and Xylazine (at dose rate 70 mg/kg and 5 mg/kg, respectively) injected intraperitoneally. The surgical site, that is, the mid‐thigh region of the experimental hind limb, was smoothly shaved and then a fine cut was given and the sciatic nerve was exposed. The nerve was crushed with the same pair of forceps by applying constant pressure for 15 s for every mouse. A transparent ring at the crushed site of the nerve would appear which ensured that the nerve had been crushed (Aziz et al., 2019; Imran et al., 2019; Rasul et al., 2019). Then the skin was sutured with 2–4 stitches and pyodine was put on the wound to avoid infection.

### 2.4 Study design

After inducing the sciatic nerve mechanical crush, all animals were equally grouped (*n* = 8) as follows: The control group was offered a daily rodent chow, and the *C. procera* treatment groups were given *C. procera* leaves mixed in rodent chow at the dose of 50 mg/kg, 100 mg/kg, and 200 mg/kg, respectively. All treatment groups were offered their respective diet from the day of nerve crush until the end of the experiment. On the last day of the study period, all animals were sacrificed after giving deep anesthesia. Blood was collected to separate serum for biochemical analyses and skeletal muscles from both hind limbs were dissected for muscle mass analyses.

### 2.5 Behavioral analyses

#### 2.5.1 Thermoceptive analysis

The hot plate analysis is a well reported behavioral parameter to assess sensory functions. It was performed by adopting the protocol given in earlier studies (Razzaq, Ahmad, et al., 2020). Briefly, the experimental hind paw (Ipsilateral) was exposed to the hot surface of the hot plate (SCILOGEX MS7‐H550‐S LED digital 7×7 Hotplate stirrers) adjusted at 56 ± 2°C and the time taken to respond (jerk, abrupt withdrawal from hot surface or paw licking) by the mouse, on exposure to the hot surface, was observed as paw withdrawal latency. The mouse was removed immediately from the hot plate upon responding. A total of three readings were taken with the time lap of 2 min in successive readings. For the mouse which showed no response until 30 s (cut off time) was removed from the hot surface to avoid thermal injury (Aziz et al., 2019; Imran et al., 2019; Rasul et al., 2019).

#### 2.5.2 Nociceptive analysis

The pinprick test is another way to assess the sensory functions retrieval after nerve injury induction. It was done by adopting the protocol as given by Chen et al., 2017. Briefly, the mouse was kept on a wire mesh cage and the lateral part of the plantar surface of its hind paw was divided into 5 areas hypothetically. Each area was pinpricked gently by an Austerlitz insect pin having size “000” and swift removal of the paw was observed as a positive response. The test was applied from the heel (A) to the most lateral toe (E) of the experimental paw. The area was graded “1” for the positive response and “0” for no response (Chen et al., 2017). Thus, a mouse showing a positive response on all 5 sites was given a score of 5 which showed a fully functional sensory response. While score, less than 5 would show partial recovery, and a score “0” means complete functional loss.

#### 2.5.3 Walking track analysis

Sciatic functional index (SFI) is a mathematical way to assess motor functions in rodents. The retrieval in motor functions after sciatic nerve crush was assessed by calculating the SFI following walking track analysis by adopting the protocol published in previous studies (Komirishetty et al., 2016). Briefly, the hind limbs of mice were painted with nontoxic ink. Thereafter the mice were allowed to walk on a 50 cm wooden track wrapped with white paper. It gave paw prints on paper. The clearer prints were recorded for further observation and the following formula had been applied to measure the SFI:

SFI=(‐38.3×‐EPL‐NPL/NPL)+(109.5×ETS‐NTS/NTS)+(13.3×EIT‐NIT/NIT)‐8.8

Here, the PL is the print length from the heel to the tip of third toe, IT is the intermediate toe spread, that is, the distance b/w the fourth and the second toe and TS is the Toe spread (distance between the first and fifth toe).

#### 2.5.4 Grip strength analysis

Grip strength assessment is a reliable way to quantify the motor function retrieval following the sciatic nerve crush injury. The gripping force of both hind limbs (ipsilateral and contralateral to lesion site) was measured individually for every mouse by the grip strength meter (Bioseb, Chaville, France) following the protocol given in earlier studies. Three readings with a gap of 1–2 min were taken and an average was calculated to take the final value (Hussain et al., 2013; Razzaq, Ahmad, et al., 2020).

### 2.6 Muscle weight analysis

Upon the loss of connection between muscle and nerve following injury, muscular atrophy begins which results in muscle mass loss. So, muscle mass assessment presents a way to assess the extent of muscle atrophy which is a major obstacle in the way of retrieving functional recovery. The soleus muscle and the tibialis anterior (TA) muscle from both hind limbs (ipsilateral and contralateral to injury site) were dissected out and harvested to measure the muscle weight by digital weighing balance. The muscle mass ratio was calculated by dividing the muscle weight of the ipsilateral side to that from the contralateral side of the same animal. The mean ratio for every group was calculated and then compared among groups to analyze functional retrieval (Tuffaha et al., 2016).

### 2.7 Biochemical analyses

#### 2.7.1 Total antioxidant capacity (TAC)

The antioxidant capacity is the ability of the living body's protective system to combat free radicals generated as a result of different pathophysiological processes going on in the body. An optimal antioxidant bearing capacity enables the body to resist various diseases. The TAC had been measured by following the protocol given by Erel, 2004 to assess the antioxidative capability in serum samples of all experimental mice after their dissection, which has been expressed in mM of vitamin C Eq. /L (Erel, 2004; Rubio et al., 2016).

#### 2.7.2 Total oxidant status (TOS)

The level of oxidative stress is known to be correlated with the extent of TOS within the living system. By using this test, the overall status of oxidative stress is evaluated. This was done by adopting the protocol mentioned in previous studies (Aziz et al., 2019; Erel, 2005). This assay works on the oxidation of o‐Dianisidine ferrous ion into ferric ion due to the presence of oxidants in the biological sample. Then xylenol orange makes a complex with ferric ions in the acidic environment which imparts color. The color intensity (indicating the oxidant status of the sample) was measured by using the spectrophotometer. For calibration, H_2_O_2_ was used as the standard and the results were expressed in terms of µM H_2_O_2_ Eq. /L (Erel, 2005).

#### 2.7.3 Paraoxonase activity

Paraoxonase (PON‐1) is a hydrolytic enzyme with an intrinsic ability to armor the living system against lipid peroxidation (Serdar et al., 2006). Its activity depends upon the degree of enzymatic degradation of paraoxon into the p‐nitrophenol in the presence of PON‐1. The standard reagent was prepared by mixing paraoxon (2 mM/L), Calcium chloride (2 mM/L), and tris HCL (0.1 M/L). Principally, the formation of p‐nitrophenol results in the color production which acts as an indicator of the enzymatic activity of PON‐1. The absorbance was measured at 405 nm wavelength at room temperature by spectrophotometer and enzymatic activity was calculated by following the already established protocol (Elkiran et al., 2007; Haagen & Brock, 1992).

#### 2.7.4 Arylesterase activity

Arylesterase (ARE) is a thiol enzyme, found to play a role in diminishing oxidative stress (Erdem et al., 2010). Assessment of ARE in biological samples gives a clue about the status of the antioxidative capability of the system. To evaluate ARE activity, Phenyl acetate was used as a substrate in the reaction solution. During this reaction, the phenylacetate was catalyzed and converted into phenol in the presence of ARE. The conversion rate was taken as a direct measure of the activity of the enzyme. This test was done by mixing the phenylacetate (2.0 mM/L) with methanol (40%) for making the stock solution. The other constituents of this solution comprised of 0.1 M/L Tris‐HCL Buffer (8.0 pH) and calcium chloride (2.0 mM/L). The absorbance was measured at 270 nm wavelength at room temperature by spectrophotometer and ARE activity was calculated by following the already established protocol (Elkiran et al., 2007; Haagen & Brock, 1992).

#### 2.7.5 Random glycemic level

The random glycemic level was observed before and after nerve injury induction to analyze the impact of glucose on aggravating the neurotic events at the nerve injury site. It is a common observation that the hyperglycemic condition in the body delays the healing pathways after injuries. The glycemic level in all groups was measured by taking a drop of blood from the tail of the mouse on a glucometer strip and this was assessed by using a glucometer (Accu‐Check) in the same way as mentioned in previous studies (Asmat et al., 2016; Razzaq, Hussain, et al., 2020).

### 2.8 Statistical analysis

All results were recorded as mean ± standard error of the mean (SEM) and were analyzed using GraphPad prism 8.4.2. Analysis of variance (ANOVA) was applied along with Tukey's multiple comparisons test to compare the means among multiple groups at different time points. A value of *p* < .05 was considered statistically significant for all observations.

## 3 RESULTS

### 3.1 Effect of *C. procera* leaves on Bodyweight and Diet consumption

**FIGURE 1:**
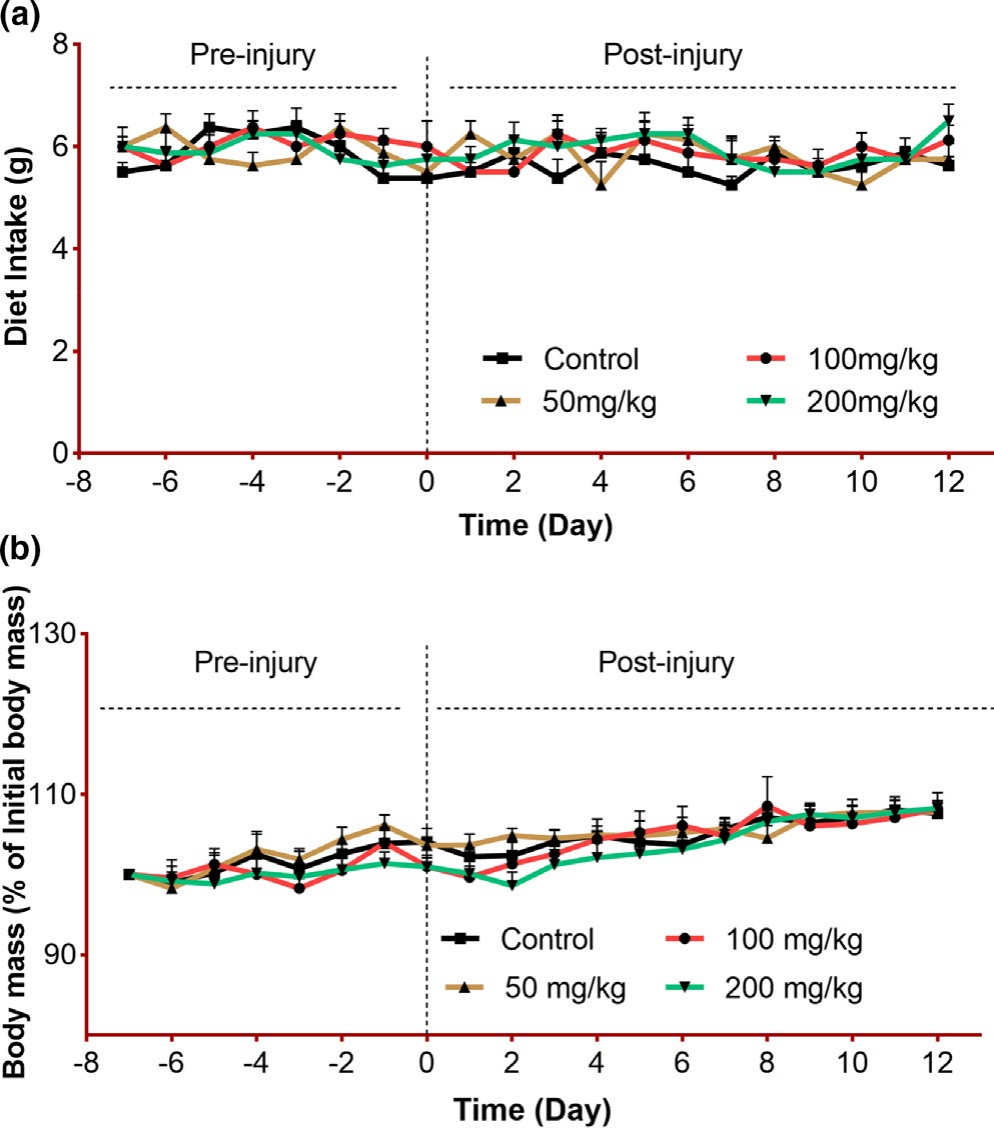
Effect of *C. procera* on diet intake and body mass: (a) Time course of diet intake in mice fed on normal chow (control, *n* = 8) or *C. procera* containing chow (50 mg/kg: *n* = 8, 100 mg/kg: *n* = 8, 200 mg/kg: *n* = 8) throughout the study period. *C. procera* containing chow was offered to treatment groups from the day of sciatic nerve crush to the end of the experiment. Two‐way repeated measure ANOVA showed a non‐significant effect of time (*F*(19, 532) = 1.24, *p* = .218), a nonsignificant effect of diet (*F*(3, 28) = 2.25, *p* = .104) and a nonsignificant interaction between factors (*F*(57, 532) = 0.833, *p* = .802). (b) Time course of body mass of mice as in (a). Body mass is expressed as a percentage of initial mass at day −7 per individual. Two‐way repeated measure ANOVA showed a significant effects of time (*F*(19, 532) = 15.9, *p* < .001), nonsignificant effect of diet (*F*(3, 28) = 0.589, *p* = .627), and a nonsignificant interaction between factors (*F*(57, 532) = 0.698, *p* = .954). Tukey's multiple comparisons test showed nonsignificant differences among all groups at all time points for (a) and (b)

The diet intake and body weight of all groups were measured throughout the study. It was observed that the diet consumption of mice was not affected after nerve injury induction and also the addition of *C. procera* at all given doses in rodent chow did not change the diet intake pattern in mice (Figure 1a). The data for diet intake analysis appeared to be nonsignificant statistically (*p* = .802). Similarly, the comparison of body mass percentage among all groups was also found to be nonsignificant (*p* = .954) during the whole time period of the experiment (Figure 1b).

Since the addition of any plant powder can alter the animals' feeding pattern either due to smell or change in taste. Therefore, body weight and diet intake analyses were conducted to figure out the impacts of adding crude leaves in the diet on animals' normal diet intake patterns. All selected doses (50, 100, and 200 mg/kg) of *C. procera* crude leaves were considered on the basis of previous studies and are reported to be nontoxic without any adverse impact on animals (Meena et al., 2011). A study conducted in sheep revealed hepatotoxic and cardiotoxic impact of c.procera leaves at the dose of 30 g/kg and 60 g/kg (de Lima et al., 2011). However, the administration of 200 mg/kg dose of *C. procera* leaves extracts (aquous, ethanol and chloroform) in rats with CCl4‐induced hepatotoxicity showed a decrease in Acid phosphatase, Alkaline phosphatase, Aspartate aminotransferase, Alanine aminotransferase, Total protein, Albumin, and total bilirubin levels which implicates its hepatoprotective effects at the given dose (Alrheam & Alrheam, 2015).

In the current study, no adverse event or mortality was observed at all given doses in any group. Thus, all selected doses were found to be safe. Nonsignificant findings among all groups suggest that the addition of *C. procera* leaves in the diet did not change the eating pattern of the mice and a consistent rise in body weight with nonsignificant differences among all groups showed an unaltered metabolic pattern in all mice. These findings are also following the observations published in a recent study showing the impact of *Moringa oleifera* on escalating functional recovery in the mouse model of PNI (Razzaq, Ahmad, et al., 2020).

### 3.2 Effect of *C. procera* leaves on motor activity

**FIGURE 2:**
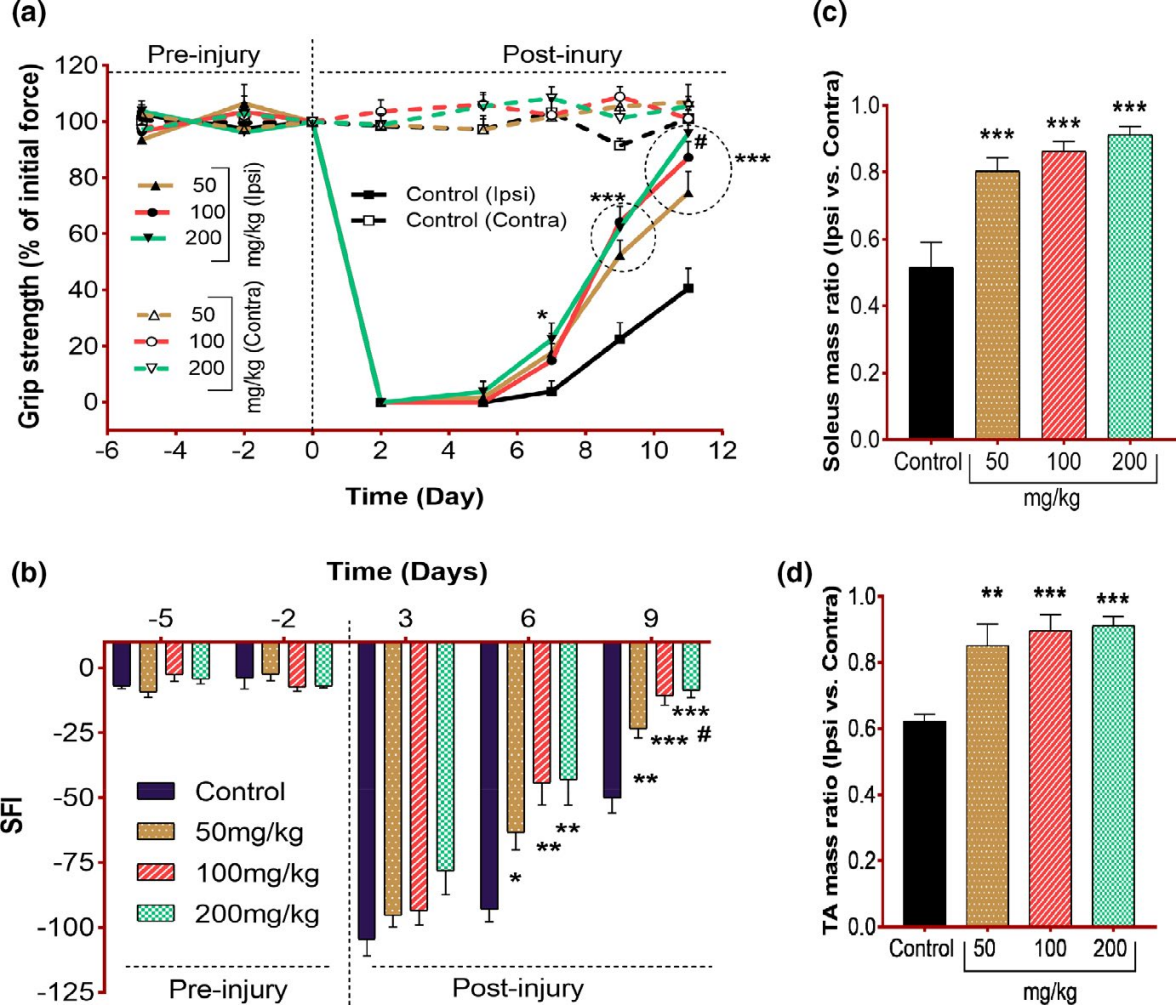
Effect of *C. procera* on motor functional recovery after nerve injury. (a) Time course of muscle grip strength in mice fed on normal chow (Control *n* = 8) or *C. procera* containing chow (50 mg/kg: *n* = 8, 100 mg/kg: *n* = 8, 200 mg/kg: *n* = 8). Measurements were obtained from hind limbs contralateral (dotted lines) and ipsilateral (solid lines) to the lesion. Grip strength is expressed as a percentage of average of initial force developed at day −5 and −2 per individual. Two‐way repeated‐measure ANOVA showed highly significant effects of time (*F*(7, 392) = 234.4, *p* < .0001) and diet (*F*(7, 56) = 190.9, *p* < .0001), and a significant interaction between factors (*F*(49, 392) = 38.28, *p* < .0001). Tukey's multiple comparisons revealed significant differences (ipsilateral side) between control versus. 200 mg/kg (*p = .04) on day 7 post‐injury and control versus. 50, 100 and 200 mg/kg (***p < .0001) on day 9 and day 11 post‐injury. A significant difference (#*p* = .02) between 50 mg/kg versus. 200 mg/kg has also been observed on day 11 postinjury. (b) Time course of the sciatic functional index (SFI) of mice as in (a). Two‐way repeated‐measure ANOVA showed significant effects of time (*F*(2.45, 61.2) = 278, *p* < .0001) and diet (*F*(3, 25) = 12, *p* < .0001), and a significant interaction between factors (*F*(12,100) = 5.91, *p* < .0001). Tukey's multiple comparison test revealed significant differences between control versus. 50 mg/kg (*p = .02), control versus. 100 mg/kg (**p = .001), control versus. 200 mg/kg (**p = .005) on day 6 post‐injury, control versus. 50 mg/kg (*p = .01), control versus. 100 mg/kg (***p = .007), control versus. 200 mg/kg (***p = .0006) and 50 mg/kg versus. 200 mg/kg on day 9 postinjury. (c) Comparison of Soleus mass of mice as in (a). Measurements are expressed as a mean muscle mass ratio between hind limbs ipsilateral and contralateral to the lesion. Ordinary one way ANOVA (*F*(3, 28) = 14.18, ***p < .0001) along with Tukey's multiple comparisons test revealed significant differences between control versus. 50 mg/kg (***p = .0009) control versus. 100 mg/kg (***p < .0001) and control versus. 200 mg/kg (***p < .0001). (d) Comparison of Anterior tibialis muscle mass of mice as in (a). Measurements are expressed as a mean muscle mass ratio between hind limbs ipsilateral and contralateral to the lesion. Ordinary one way ANOVA (*F*(3, 28) = 9.13, ***p = .0002) along with Tukey's multiple comparisons test revealed significant differences between control versus. 50 mg/kg (**p =.006) control versus. 100 mg/kg (***p = .0009) and control versus. 200 mg/kg (***p = .0005)

Sciatic nerve contains both sensory and motor neurons which innervate the lower region of the body. Compression of the sciatic nerve results in loss of conduction to the skeletal muscles, which eventually results in loss of motor function of that limb (Hussain et al., 2020). Motor function restoration happens upon revival of nerve impulses (Carvalho et al., 2020). In the current scenario, a significant restoration in the motor functions was observed in terms of SFI measurement and the grip strength force (% of initial force) in all treatment groups as compared to the control (Figure 2a,b). Treatment groups had shown a prominent difference (earlier restoration of gripping force of affected paw to grip the wire of grip strength meter) relevant to control on day 7 postinjury, particularly the 200 mg/kg group appeared to be suggestively effective (*p* = .04). On subsequent time points (day 9 and 11 postinjury), all treatment groups showed meaningfully enhanced gripping strength differences (*p* = .0001) as compared to the control. However, the 200 mg/kg group also showed weighty differences relevant to 50 mg/kg group on day 11 postinjury (*p* = .02). Data of SFI indicate the prominent improvement in pattern of walking in animals of treated groups. Here the 100 and 200 mg/kg groups appeared to be highly effective in normalizing the SFI values on day 6 (*p* = .001) and day 9 (*p* = .006) postinjury. These findings indicate the potential of *C. procera* leaves in escalating motor functional recovery.

The earlier revival of motor function is associated with axonal growth (Ma et al., 2011). Natural products are found to enhance neuronal growth (An et al., 2018). Flavonoids have been reported to enhance the glycoxalate pathway (antioxidant defense system of neuronal tissues of the brain) and prevent the cerebral tissues from degeneration (Frandsen & Narayanasamy, 2017). *C. procera* leaves have been reported to possess active constituents such as alkaloids, flavonoids, tannin, saponin, terpenoids, cardiac glycoside, and phenols (Falana & Nurudeen, 2020; Shobowale et al., 2013). The earlier motor function recovery indicates toward the possession of neuroregenrative potential of leaves. The tibialis anterior and soleus muscles are skeletal muscles in the hind limbs, innervated by the sciatic nerve. These muscles are found to be involved in walking, running, inward, and outward flexing of the foot (Razzaq, Hussain, et al., 2020). Upon sciatic nerve crush, motor endplates of these muscles get denervated and the muscle stops working. Meanwhile, they start shrinking due to no innervation. So we measured and compared their muscle mass from ipsilateral and contralateral hindlimbs after dissecting them out from the mice to find the recovery in muscle mass of the affected side. The administration of fennel seeds and extract (Imran et al., 2019; Maqbool et al., 2020), *Neurada procumbens* (Rasul et al., 2019), crude Moringa leaves (Razzaq, Hussain, et al., 2020) in animal model of peripheral nerve injury showed comparatively improved muscle mass ratio which had been reported to be due to the positive impact of treatments on enhancing neuronal growth. Another study showing the implication of cytokine cocktail's effect on axonal regeneration in the mouse model reported the potential recovery in muscle mass under the influence of treatment (Maki et al., 2021). In the current findings, the muscle mass for both muscles was increased prominently (almost close to its contralateral hindlimb) in the treatment groups as compared to the control (Figure 2c). *C. procera* leaves at all doses had shown significantly increased value of muscle mass ratio (*p* = .0005). The increased muscle mass in the treatment groups supported the findings as observed while motor function analyses via the grip strength and SFI measurement. These findings clearly support the idea that *C. procera* has the potential to speed up nerve regeneration and restore nerve functions before the beginning of muscular atrophy.

### 3.3 Effect of *C. procera* leaves on sensory threshold activity

**FIGURE 3:**
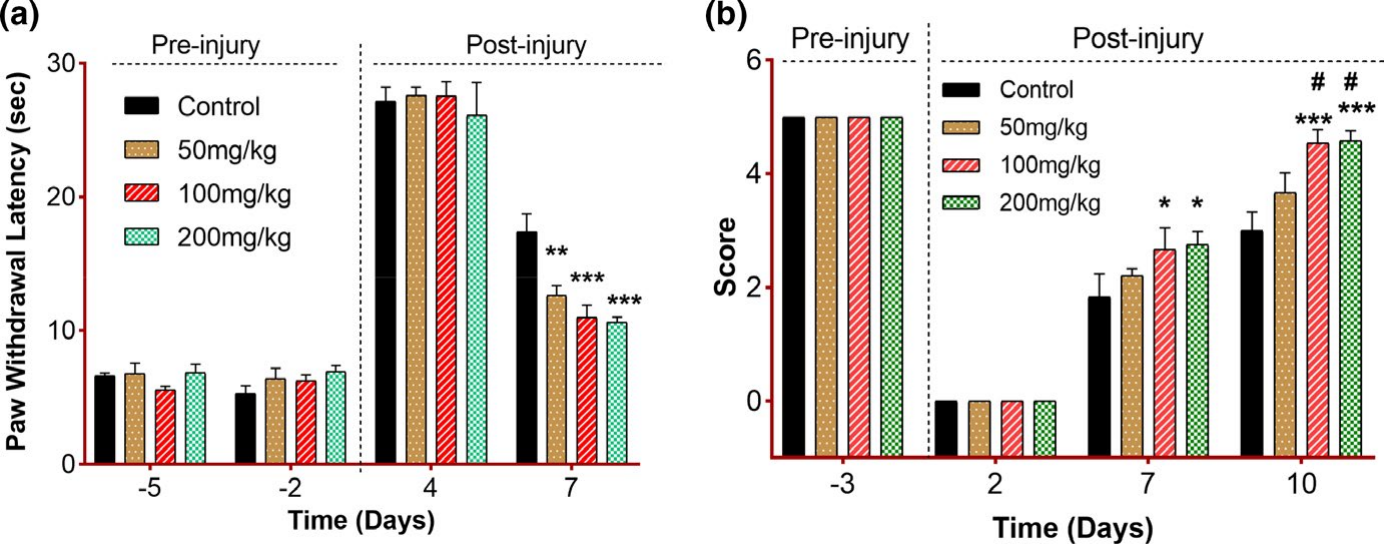
Effect of *C. procera* on sensory threshold retrieval after sciatic nerve injury. (a) Paw withdrawal latency in response to thermal stimulation in mice fed on normal chow (Control *n* = 8) or *C. procera* containing chow (50 mg/kg: *n* = 8, 100 mg/kg: *n* = 8, 200 mg/kg: *n* = 8). Measurements were obtained at different time points before and after injury induction. Two‐way repeated measure ANOVA showed a significant effect of time (*F*(3, 81) = 405, *p* < .001), a nonsignificant effect of diet (*F*(3, 27) = 2.41, *p* =.09), and a significant interaction between factors (*F*(9, 81) = 3.28, *p* = .002). Tukey's multiple comparison test revealed significant differences between control versus. 50 mg/kg (**p = .004), control versus. 100 and 200 mg/kg groups (***p < .001) on day 7 postinjury. (b) Paw withdrawal score in response to pinprick stimulation in mice as in (a). Two‐way repeated measure ANOVA showed significant effects of time (*F*(3, 84) = 531.2, *p* < .0001) and diet (*F*(3, 28) = 5.24, *p* = .005), and a significant interaction between factors (*F*(9, 84) = 3.821, *p* = .0004). Tukey's multiple comparison test revealed significant differences between control versus. 100 mg/kg (*p = .02), control versus. 200 mg/kg (*p = .01) on day 7 post‐injury and control versus. 100 and 200 mg/kg (***p < .0001) on day 10 postinjury. A significant difference between 50 mg/kg versus. 100 mg/kg (#*p* = .017) and 50 mg/kg versus. 200 mg/kg (#*p* = .012) has also been observed on day 10 postinjury

Following the sciatic nerve injury, the retrieval of sensory functions was measured by the hot plate and pinprick test (Figure 3a,b). Both analyses are always a good means of measuring sensory threshold retrieval in mice (Deuis et al., 2017). A highly significant improvement (quick response to thermal stimulus) was observed in all treatment groups as compared to the control. Both 100 mg/kg and 200 mg/kg groups appeared to be equally effective in showing a statistically significant decrease (*p* = .001) in paw withdrawal latency from the hot surface on day 7 postinjury while the 50 mg/kg group appeared to be significantly effective at *p* = .004 in quickening the paw withdrawal from the thermal stimulus. Similarly, sensory threshold assessment by the pinprick test was observed and all treatment groups appeared to be effective in showing comparatively higher scores against the pinprick stimulus. However, the 100 and 200 mg/kg groups appeared to be significantly effective (*p* = .01) on day 7 postinjury and highly effective (*p* = .0001) on day 10 postinjury. Both groups also showed statistically significant improvement in results (#*p* = .01) as compared to the 50 mg/kg group. These findings validate the effectiveness of *C. procera* in accelerating the sensory threshold following the sciatic nerve injury.

As discussed earlier about the mixed nature of the sciatic nerve, a mechanical insult in it also results in the loss of sensory functions along with motor functions (Perussi Biscola et al., 2016). A statistically prominent sensory threshold retrieval, as assessed by the hot plate test (thermoception) and the pinprick test (nociception) in treatment groups implicates toward the fact that *C. procera* had significantly decreased the paw withdrawal latency from the hot surface and also gave a full score following nociceptive stimulus via the pricking pin on the dorsolateral surface of the ipsilateral paw, suggests the ameliorative effect of this plant on accelerating axonal regeneration and ultimately functional recovery.

### 3.4 Effect of *C. procera* leaves on systemic indices

In addition to behavioral assessments, biochemical assessments were also done to validate the effectiveness of this plant in promoting axonal regeneration. These tests also give some clues about the possible mechanism involved behind this improvement. For this, the glycemic level was measured and compared. Statistically significant reduction in glycemia following *C. procera* containing diet consumption reflects the hypoglycemic effect of this plant. Pathological events that happened following injury may alter the blood glucose level. Hyperglycemic conditions further exacerbate the inflammatory processes and tissue necrosis notably due to increased oxidative stress in the body (Asmat et al., 2016). *C. procera* is already well known for its antidiabetic/ hypoglycemic effects in various studies (Kazeem et al., 2016; Mali et al., 2019). The finding of the current study supports the fact that *C. procera* exhibits potential to decrease the glucose level in the blood up to the normal level.

**FIGURE 4:**
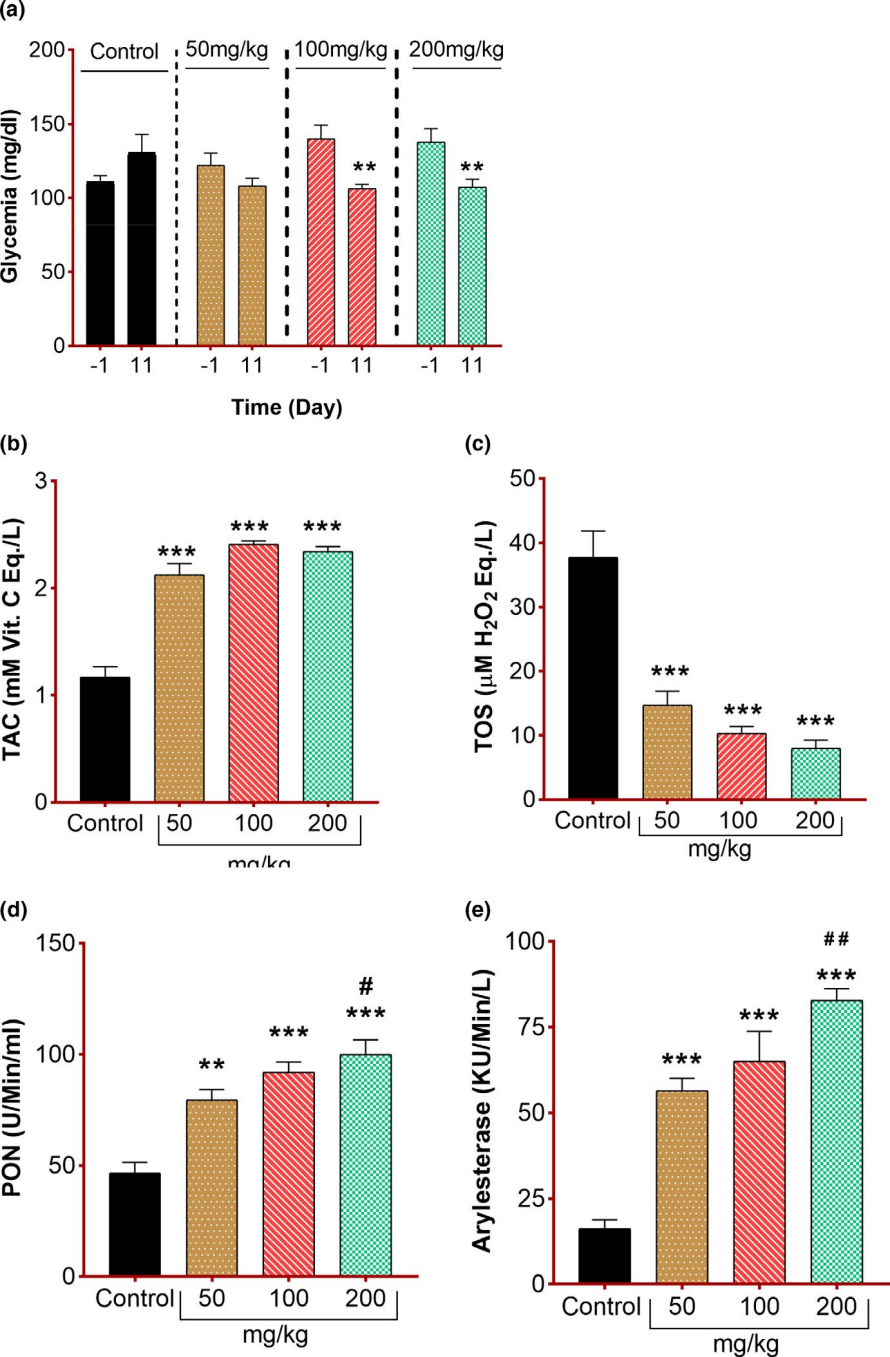
Effect of *C. procera* on systemic indices (a) Glycemia measured in mice fed on normal chow (Control *n* = 8) or *C. procera* containing chow (50 mg/kg: *n* = 8, 100 mg/kg: *n* = 8, 200 mg/kg: *n* = 8). Measurements were obtained 1 day before sciatic nerve crush and after 11 days of functional recovery subsequent to sciatic nerve crush in all groups. Two‐way repeated measure ANOVA showed nonsignificant effects of diet (*F*(3,28) = 0.34, *p* = .7) and significant effect of time (*F*(1,28) = 11.67, *p* = .002), and a significant interaction between factors (*F*(3, 28) = 8.26, *p* = .0004). Sidak's multiple comparison test revealed a significant reduction in the glycemia level from values observed before initiating the treatment for 100 and 200 mg/kg (**p = .002) groups. (b) Total antioxidant capacity (TAC) of mice as in (a). Ordinary one‐way ANOVA showed highly significant effect of *C. procera* containing diet (***p < .0001) in elevating the TAC values. Tukey's multiple comparisons test revealed significant differences between control versus. 50, 100 and 200 mg/kg groups (***p < .0001). (c) Total oxidant status (TOS) of mice as in (a). Ordinary one‐way ANOVA showed highly significant effect of *C. procera* containing diet (***p < .0001) in reducing the TOS values. Tukey's multiple comparisons test revealed significant differences between control versus. 50, 100 and 200 mg/kg groups (***p < .0001). (d) Paraoxonase activity measurement in mice as in (a). Ordinary one way ANOVA showed highly significant effect of *C. procera* containing diet (*F*(3,28)=19.8, ***p < .0001) in increasing Paraoxonase activity. Tukey's multiple comparisons test revealed significant differences between control versus. 50 mg/kg (***p = .0007), 100 mg/kg (***p < .0001) and 200 mg/kg (***p < .0001) and also 50 mg/kg versus. 200 mg/kg (^#^ *p* = .04). (e) Arylesterase activity measurement in mice as in (a). Ordinary one way ANOVA showed highly significant effect of *C. procera* containing diet (*F*(3,28) = 29, ***p < .0001) in increasing Arylesterase activity. Tukey's multiple comparisons test revealed significant differences between control versus. 50 mg/kg (***p <.0001), 100 mg/kg (***p < .0001) and 200 mg/kg (***p < .0001) and also 50 mg/kg versus. 200 mg/kg (^##^ *p* = .006)

Hyperglycemia in the body is found to exacerbate the pathological events happening at the injury site (Kwai et al., 2016; Sala et al., 1999). So, the optimal glycemic level in the body is a prerequisite to healing injuries in time. In this context, the glycemic level was also recorded and compared before and 11 days after injury induction among all groups (Figure 4a). It was observed that all treatment groups kept the glycemic level up to normal. However, reasonably higher level of glucose was observed in mice fed on normal chow (control group). *C. procera* leaves at the dose of 100 and 200 mg/kg lowered the glycemic level significantly (*p* =.002). Antidiabetic effect of the *C. procera* plant particularly its leaves is well known in previously published literature (Kazeem et al., 2016). The findings of this experiment support those pieces of evidence regarding the glucose‐lowering capability of *C. procera* plant.

Oxidative stress is one of those major hallmarks, contributing prominently in exacerbating the pathological phenomena happening at the injury site (Wang et al., 2015). Following nerve injury, cascades of reactions (i.e., mitochondrial dysfunctions, demyelination, neuroinflammation, and apoptosis (Hussain et al., 2020)) happen which result in the generation of oxidants that worsen the injury and delay the regenerative processes (Al‐Nimer et al., 2012; Areti et al., 2014). *C. procera* is a well‐reported antioxidant as well as an antiapoptotic agent (Nadeem et al., 2019; Sayed et al., 2016). Though this plant is renowned for its antioxidant properties yet no data is available until now about its role in healing nerve injuries and improving nerve regeneration. After decapitation, the measurement of TAC and TOS in serum samples of animals was conducted to elucidate the possible mechanism behind improved functional recovery and the results revealed a highly significant impact of *C. procera* leaves on reducing oxidative stress (i.e., improved TAC values and reduced TOS values).

A highly significant increase (*p* < .0001) in TAC values in all treatment groups as compared to the control was observed (Figure 4b) which confirms the possession of a highly effective antioxidant capacity of this *C. procera* leaves. Similarly, in the case of sciatic nerve injury, the level of free radicals increases which in turn increases the TOS level. A highly significant reduction (*p* < .0001) in TOS values in all treatment groups as compared to control was observed (Figure 4c). The same trend of effectiveness was observed in all *C. procera* treated groups, which showed significantly higher activity of oxidative stress‐related enzymes, that is, PON‐1 and ARE (Figure 4d,e).

A highly significant rise in the activity of these enzymes in the treatment groups provide profound evidence of *C. procera's* oxidative stress combating role in the treatment groups. These findings not only support the antioxidative potential possessed by *C. procera* leaves but also give an idea that significantly improved observations depicting enhanced functional recovery would be due to antioxidant effects, which were contributed by this plant. Based on these findings and knowledge, it can be suggested that to reduce the development of pathological pathways worsening the PNI, antioxidants might be of potent choice.

## 4 DISCUSSION

Despite various advancements in the present era, mankind has been facing different types of afflictions. Particularly, in the field of medical sciences, one of the major afflictions is to control the continually increasing cases of life‐long disability or physical dependency due to nerve injuries, which develop as a result of different kinds of accidents particularly roadside motor vehicles collisions, gunshots, and sudden fall (Kouyoumdjian et al., 2017). Unfortunately, these incidents are among the major causative agents behind PNI development, predominantly in over‐populated underdeveloped countries like Pakistan, where the traffic control system is already compromised (Mushtaq et al., 2021). Though medical science contributes significantly in controlling the variety of health issues by introducing a huge number of therapeutic ways, yet some complications still need to be addressed on an urgent basis to get rid of them. PNI is one such complication to be faced. A variety of therapeutic methods have been adopted by physicians to control pathetic consequences following nerve injuries yet, they remain unsuccessful in completely reviving the normal sensorimotor functions in most cases. The reason behind this failure is the complicated pathophysiology of PNIs which still need to be explored. Although it is an innate ability of a damaged nerve to regenerate itself, the process of regeneration to reinnervate the distal organ is too slow and time‐consuming to accomplish. This process further has to face a delay because of parallel ongoing pathological changes in the target muscle (muscle starts atrophying). So, seeking an alternative therapy to speed up the process of regeneration and reinnervation of the damaged axon to achieve an impeccable and complete functional recovery is the dire need of the hour and the main objective behind designing this study. Medicinal plants have always remained a favorite option for physicians to treat diseases since ancient times. *C. procera* (member of family Apocynaceae) is a tropical plant of high significance and medicinal importance and used in curing diseases of medical concern over the past years (Paul et al., 2018). This plant attracted our attention because of its reported effectiveness as an antioxidant, anti‐inflammatory, and neuroprotectant (Mali et al., 2019). This plant is also found to be potentially effective in clearing the beta‐amyloid proteins accumulation in neuronal tissues in Alzheimer's disease (Paul et al., 2018). Thus, it can be hypothesized that phytoconstituents hidden in this plant may also be effective in promoting the axonal regeneration accomplishment before the distal muscle gets shrunken. For this, the current experiment was planned to evaluate *C. procera* leaves as a prime step just to evaluate its role in inducing earlier functional restoration after sciatic nerve injury induction in an already established mouse model of PNI. For this, healthy adult mice were induced with a sciatic nerve mechanical crush and were offered a diet containing *C. procera* crude leaves at different doses since the day of the nerve crush. In the supervision of observations discussed above, it can be speculated that crude leaf powder of *C. procera* accelerates the motor functions recovery and regeneration of the peripheral nerve more effectively at all given doses. However, a dose of 200 mg/kg was found to be significantly more effective than that of 50 mg/kg. In contrast, differences between 100 and 200 mg/kg appeared to be nonsignificant. The desired therapeutic effects can be achieved at a dose of 100 mg/kg.

## 5 CONCLUSION

The study can be concluded in the light of all of the above‐mentioned aspects that *C. procera* (crude leaves), not only accelerated the functional recovery which has been confirmed through behavioral parameters' results but also prominently combats oxidative stress. This outcome highlights the protective effect of *C. procera* plant and encourages further evaluation. The prominently sufficient therapeutic effects can be achieved at a dose of 100 mg/kg. However, there is a dire need to investigate those bioactive compounds of *C. procera* leaves that may be responsible for these health‐promoting effects. Moreover, it would be interesting to explore all possible molecular mechanisms underlying this escalated axonal regeneration that are going to be affected by this remedy.

## CONFLICT OF INTEREST

Authors declared that they have no conflict of interest.

## ETHICAL APPROVAL

Following a careful review of the protocols utilized to conduct experiments on mice in the current study, approval was granted (ERB NO. 1986) by the Institutional Review Board (IRB) of Government College University, Faisalabad, Pakistan.

## Data Availability

The data set supporting the conclusions of this article is included in the article.
